# Diffuse neurofibroma within nevus of Ota on the left face

**DOI:** 10.1016/j.jdcr.2024.05.001

**Published:** 2024-05-10

**Authors:** Sheng Zhong, Lin Feng, Yin Yu

**Affiliations:** Department of Dermatology, Chongqing Hospital of Traditional Chinese Medicine, Chongqing, China

**Keywords:** coexistence, dermal melanocytosis, diffuse, neural crest defect, neurofibroma, nevus of Ota

## Introduction

Neurofibroma is a common tumor of nerve sheath origin, whereas nevus of Ota is a type of dermal melanocytosis. The coexistence of dermal melanocytosis and neurofibromas is rare. We present an interesting case in which the 2 conditions occurred at the same site. The diagnosis was based on the analysis of clinical characteristics, histopathology, special staining, and immunohistochemical staining results. The coexistence may represent a neural crest defect during early embryonic development.

## Case

**Fig 1:**
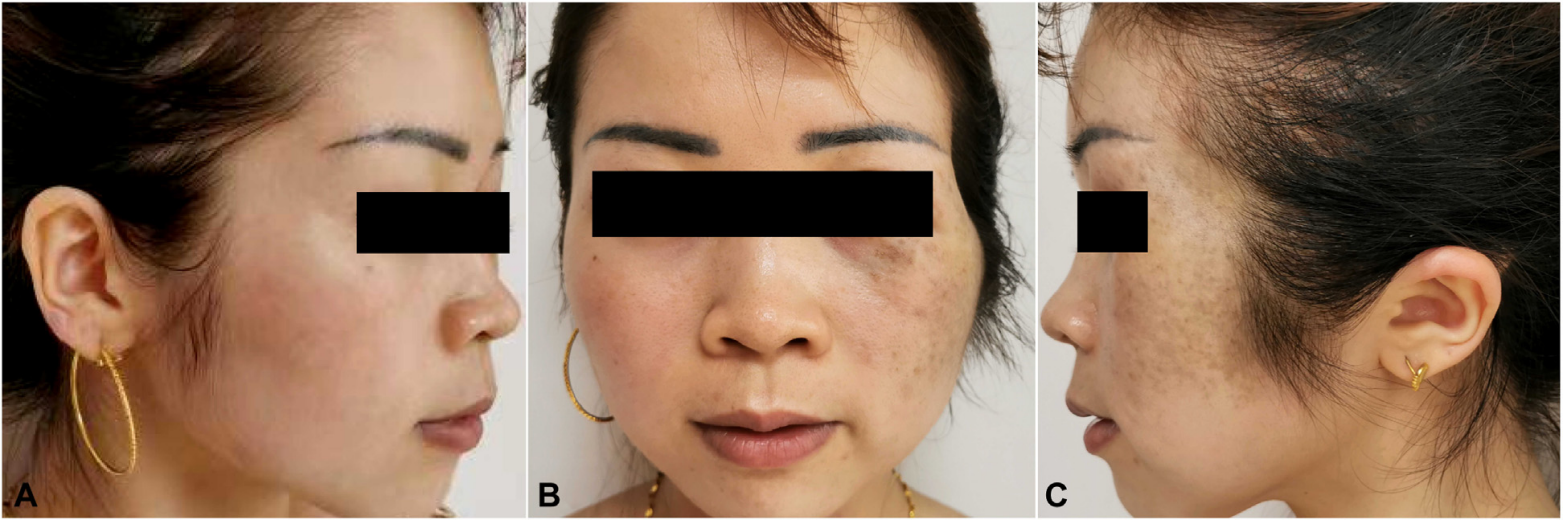
Clinical manifestation. **A**-**C,***Gray-blue* macules and sagged subcutaneous thickening on the left face of a young woman.

**Fig 2:**
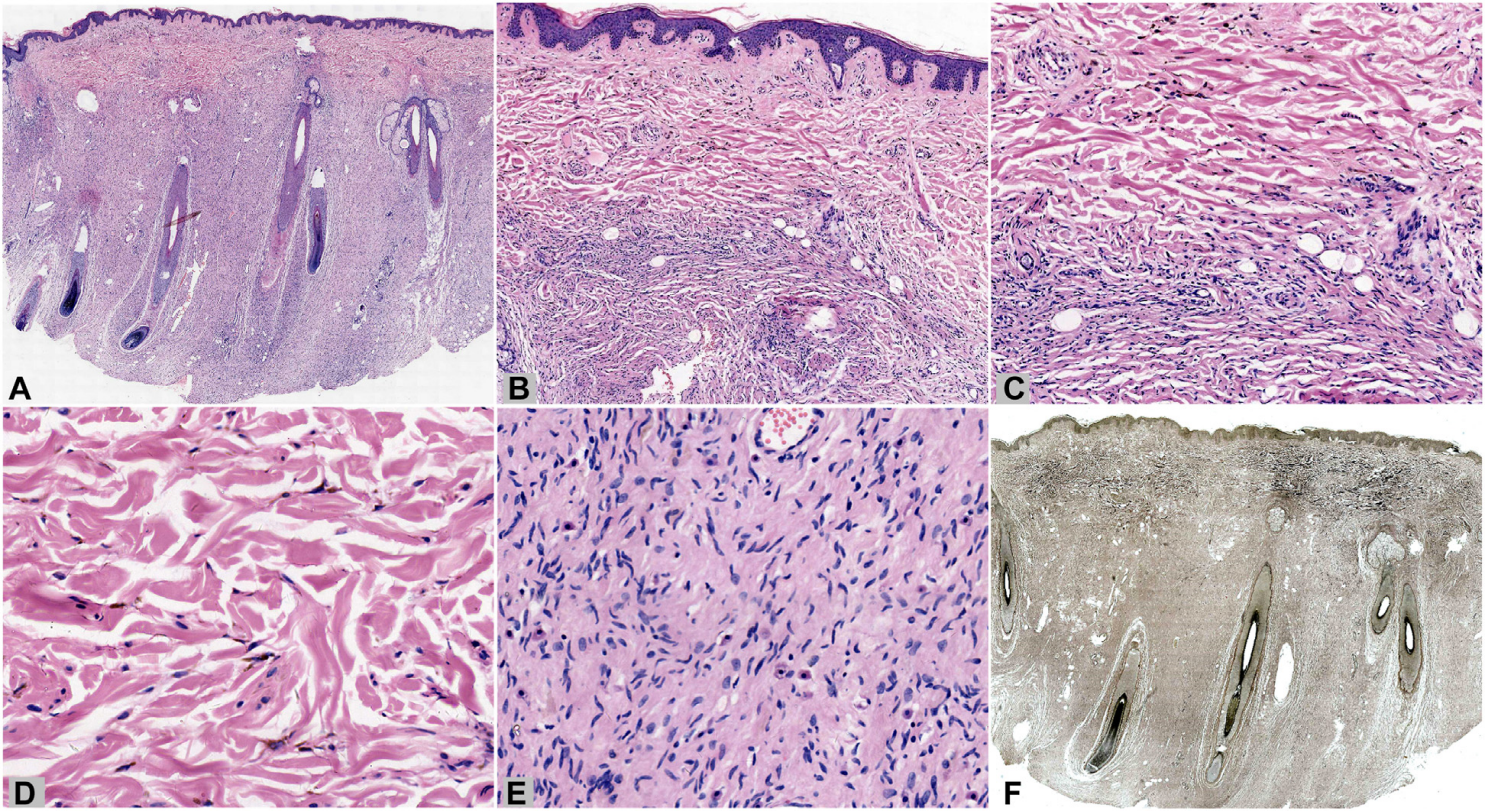
Histopathological examination and special staining. **A,** Low magnification image showing a diffuse infiltrative pattern of loose tumor mass in the deep dermis and subcutaneous fat along with scattered pigmented cells in the mid-upper dermis. (Hematoxylin-eosin stain; original magnification: ×10.) **B** and **C,** Medium magnification image showing the junction of the 2 lesions, where pigmented dendritic cells are scattered on the top and loose spindle cells are in a vortex growth pattern on the bottom. (Hematoxylin-eosin stain; (**B**) original magnification: ×50; (**C**) original magnification: ×100.) **D,** High magnification image showing pigmented, elongated, dendritic cells scattered among the collagen bundles. (Hematoxylin-eosin stain; original magnification: ×200.) **E,** High magnification image showing loose spindle cells with wavy nuclei in rich stromal components, with visible mast cells. (Hematoxylin-eosin stain; original magnification: ×200.) **F,** Reduction or loss of elastic fibers in the neurofibroma region. (Elastic-van Gieson stain; original magnification: ×10.)

**Fig 3:**
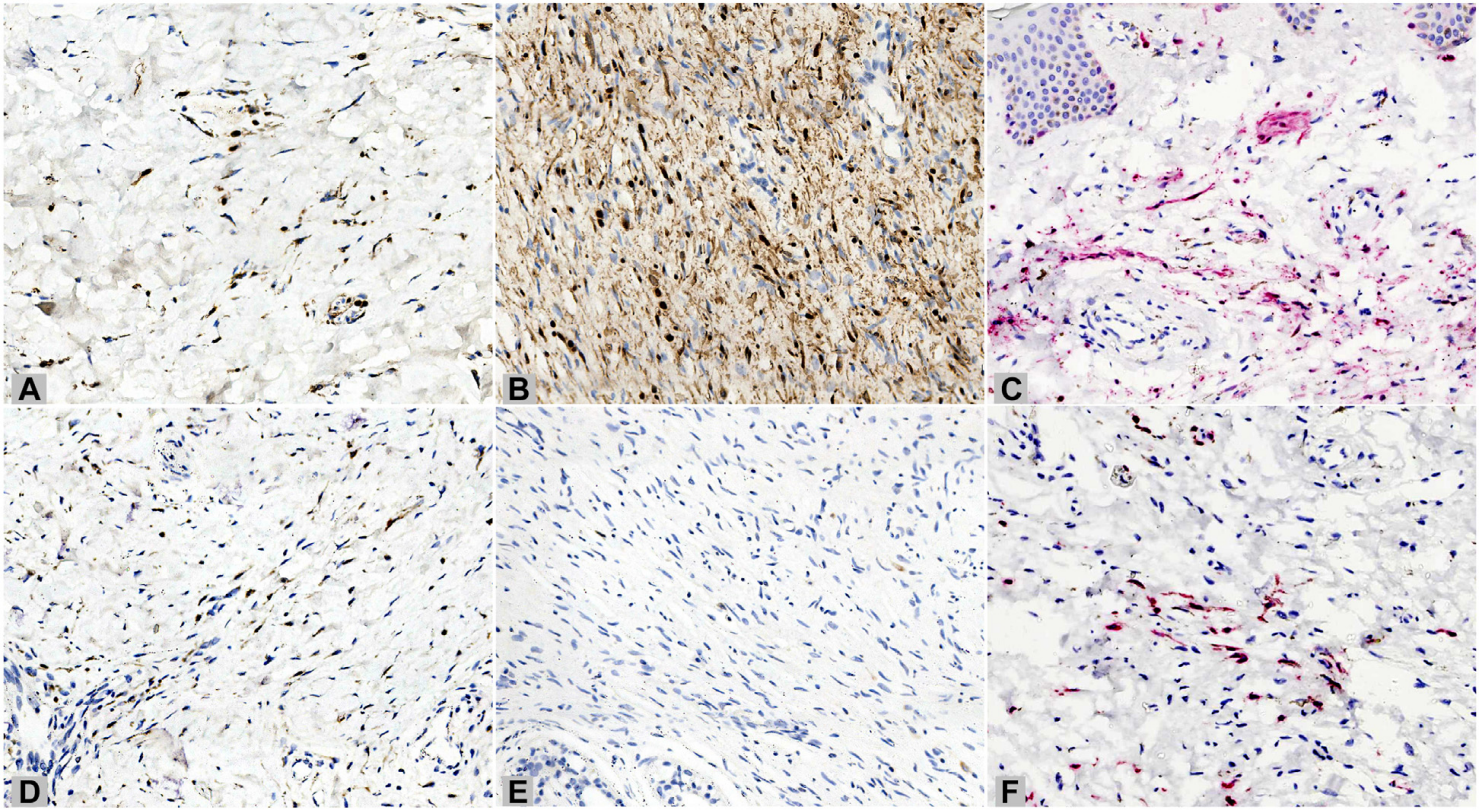
Immunohistochemical staining. **A**-**C,** Positivity for S100 in both dendritic cells (**A** and **C**) and loose spindle cells (**B**). (**A** and **B,** S100 stain; (**C**) S100 stain with red chromogen; original magnification: ×200.) **D**-**F,** Positivity for Melan-A in dendritic cells (**D** and **F**) and negativity in loose spindle cells (**E**). (**D** and **E,** Melan-A stain; (**F**) Melan-A stain with red chromogen; original magnification: ×200.)

A 31-year-old woman presented with gray-blue macules with sagging and thickening of the left face for 10 years. Both the macules and thickening appeared almost simultaneously and remained stable after gradual enlargement, without subjective symptoms. The patient had no family history, systemic disease, or corresponding symptomatic manifestations of neurofibromatosis. Physical examination revealed gray-blue macules on the left periorbital, temporal, jugal, and pre- and retroauricular regions, with sagged, soft, subcutaneous thickening without a clear boundary, where the skin could be pulled up manually (Fig 1, *A*-*C*). No café-au-lait macules, freckles, masses, or developmental abnormalities were observed in other parts of the body. Routine laboratory tests and a cranial computed tomography scan showed normal results. Histopathological examination revealed dendritic cells containing obvious pigment scattered in the upper and mid-dermis (Fig 2, *A*-*D*). In addition, loose spindle cells with wavy nuclei were found in the deeper dermis and subcutaneous fat, forming a diffuse infiltrative pattern, along with mast cells and consistent with neurofibroma (Fig 2, *A*-*C* and *E*). Elastic-van Gieson staining revealed elastic fiber reduction or loss in the neurofibroma component (Fig 2, *F*). Immunohistochemically, the dendritic cells were positive for S100 (Fig 3, *A* and *C*), SRY-box transcription factor 10, and melanoma antigen recognized by T-cells 1 (Melan-A) (Fig 3, *D* and *F*), whereas the loose spindle cells were positive for S100 (Fig 3, *B*), partially positive for SRY-box transcription factor 10, and negative for Melan-A (Fig 3, *E*). The final diagnosis, based on the clinical and pathological findings, was diffuse neurofibroma with nevus of Ota.

## Discussion

Neurofibroma is a tumor of nerve sheath origin. The skin-colored, polypoid, soft lesions of neurofibroma can occur in any part of the skin. The histopathology shows a nonencapsulated dermal or subcutaneous tumor consisting of loosely arranged spindled cells, which mainly include Schwann cells, perineurial cells, and fibroblasts. Scattered inflammatory cells, especially mast cells, can also be observed. Diffuse neurofibroma is a specific subtype of neurofibroma that generally occurs on the head, neck, or back of young people. It is clinically characterized by subcutaneous thickening with no clear boundary and histologically by a diffuse infiltrative growth pattern in uniformly collagenous stroma. Meissnerian differentiation can often serve as a histological feature.

Nevus of Ota is a type of dermal melanocytosis. It occurs mainly in darkly pigmented individuals and more frequently in women. The skin lesions are usually unilateral gray-blue macules distributed on the first 2 branches of the trigeminal nerve. Two-thirds of patients can exhibit ipsilateral scleral involvement. The color of lesions may be associated with hormonal changes. The histopathology of nevus of Ota reveals dendritic melanocytes with significant pigment scattered among collagen bundles, mostly in the upper third of the reticular dermis.

The combination of neurofibroma and dermal melanocytosis is rare. Only a few studies have reported their coexistence, but most were occurring in separate anatomic sites.^1^^,^^2^ Kim et al^3^ reported a 26-year-old Korean man with gray-to-bluish patches and an underlying neurofibroma mass on the left side of his scalp and face. It was the only case where these 2 conditions occurred at the same site, other than our case. It is currently hypothesized that as both neural cells and melanocytes differentiate from the neural crest, the combination of a neural origin tumor and dermal melanocytosis may represent a neural crest defect during early embryonic development.^4^ In early embryonic development, when migrating to target locations, neural crest cells that harbor multidirectional differentiation potential are guided via environmental cues to express specific phenotypes corresponding to particular sites. The present hypothesis is that a defect in neural crest may lead to the abnormal differentiation of excessive hyperplastic Schwann cells and perineurial cells and abnormal formation of the nerve fibers in the wrong sites, which together constitute neurofibroma. In the same sites, melanocytes that differentiate from the defective neural crest are hindered from migrating to the epidermis and are trapped in the dermis. These cells do not undergo apoptosis and then form nevus of Ota. Melanocytes and neural cells from the same source can co-express S100 and SRY-box transcription factor 10, whereas Melan-A exclusively stains melanocytes. Thus, Melan-A can be used for distinguishing melanocytes from nerve cells. The combination of staining patterns can be used as histochemical support for diagnosis.

Neurofibroma can be treated by surgical resection. Neurofibroma in neurofibromatosis type Ι, especially the plexiform or atypical variant, may have a tendency to undergo malignant change, whereas other subtypes rarely become malignant. Clinicians should be alert to painful, enlarging, or multiple skin lesions along with café-au-lait macules and other manifestations of neurofibromatosis type Ι, including ocular, skeletal, or nervous systematic alloplasia, hypertension, Lisch nodules, optic gliomas, or other extracutaneous tumors. Nevus of Ota can be treated successfully using a picosecond or Q-switched alexandrite laser. Only rare individual cases may undergo malignant transformation, such as uveal melanoma. If the pigmentation spreads to the eye, patients should be followed up carefully with ophthalmologic examination, B-scan ultrasonography, and computed tomography. Any change in the eye lesions or appearance of a subcutaneous nodule or mass necessitates pathological evaluation. Our patient exhibited stable asymptomatic skin lesions with no scleral or systemic involvement and declined treatment. The patient remains under observational follow-up.

## Conflicts of interest

None disclosed.
